# *Nocardia cyriacigeorgica* Elicits Gut Disturbances in a Leaky Gut Model of Colitis, but Not the Harmful Cascade Leading to Gut-First Parkinson’s Disease

**DOI:** 10.3390/ijms25063423

**Published:** 2024-03-18

**Authors:** João Duarte Magalhães, Emanuel Candeias, Inês Melo-Marques, António E. Abreu, Ana Raquel Pereira-Santos, Ana Raquel Esteves, Sandra Morais Cardoso, Nuno Empadinhas

**Affiliations:** 1 CNC—Center for Neuroscience and Cell Biology, University of Coimbra, 3004-517 Coimbra, Portugal; joaoduartemagalhaes7@gmail.com (J.D.M.); eu.emago@hotmail.com (E.C.); inesjose2001@gmail.com (I.M.-M.); emanuel_ramos12@hotmail.com (A.E.A.); araqpsantos@gmail.com (A.R.P.-S.); numenius@cnc.uc.pt (N.E.); 2 CIBB—Centre for Innovative Biomedicine and Biotechnology, University of Coimbra, 3004-517 Coimbra, Portugal; 3 Ph.D. Programme in Biomedicine and Experimental Biology (PDBEB), Institute for Interdisciplinary Research, University of Coimbra, 3030-789 Coimbra, Portugal; 4 Faculty of Medicine, University of Coimbra, 3004-504 Coimbra, Portugal

**Keywords:** Parkinson disease, mitochondria, bacterial infection, innate immunity, α-synuclein

## Abstract

Parkinson’s disease (PD) is a progressive neurodegenerative disorder with an unknown cause. Recent research has highlighted the importance of the gut in neuronal and immune maturation through the exchange of nutrients and cellular signals. This has led to the “gut-first PD” hypothesis, which aims to explain many of the sporadic cases and their prodromal intestinal symptoms, such as constipation and intestinal α-synuclein (aSyn) aggregation. The link between mitochondrial dysfunction and aSyn deposition is central to PD pathophysiology, since they can also trigger pro-inflammatory signals associated with aSyn deposition, potentially contributing to the onset of PD. As mitochondria are derived from ancestral alpha-proteobacteria, other bacteria may specifically target this organelle. We sought to use *Nocardia cyriacigeorgica*, a bacterium previously associated with parkinsonism, and dextran sulfate sodium (DSS) as pro-inflammatory modulators to gain further insight into the onset of PD. This study indicates that aSyn aggregation plus mitochondrial dysfunction without intestinal barrier leakage are not sufficient to trigger gut-first PD.

## 1. Introduction

Parkinson’s disease (PD), the second most common neurodegenerative disease, is characterized by a progressive impairment of movement, including postural instability, gait disturbances, and bradykinesia [1]. These symptoms are caused by the degeneration of dopaminergic neurons in the substantia nigra pars compacta (SNpc), resulting in a dopamine deficiency in the striatum and, consequently, a loss of motor control. Although the chronological progression is still debated, dopaminergic neurodegeneration is associated with the accumulation of aggregated α-synuclein (aSyn) [2]. During the prodromal phase of PD, which can begin decades before a clinical diagnosis, patients often experience a variety of symptoms. These include gastrointestinal complications, hyposmia, and sleep disorders [3]. As gastrointestinal problems, such as dysphagia, constipation, bloating, and gastroparesis, are among the most commonly reported symptoms in PD patients [4], the gastrointestinal (GI) tract has been suggested to play a pivotal role in the etiology and progression of the disease. Some studies have found that individuals in the prodromal phase of PD have higher levels of aSyn deposition in the gastrointestinal tract than age-matched controls [5]. The body-first theory emerged to explain the etiology of age-associated PD cases [6]. However, despite having some robust arguments, it fails to identify and explain the trigger for aSyn aggregation and propagation.

PD pathophysiology is characterized by a systemic inflammatory environment, with elevated levels of pro-inflammatory cytokines [7]. Increased inflammation in the gut can lead to several undesirable consequences commonly observed in PD patients, such as an increase in intestinal permeability, also known as “leaky gut” [8,9,10]. This can facilitate the translocation of bacteria or bacterial metabolites from the gut into surrounding organs and the circulatory system [11]. Several studies have reported that patients with inflammatory bowel disease (IBD) who have exacerbated gut inflammation and increased intestinal permeability are more likely to develop PD than age-matched healthy individuals [12]. Furthermore, through the analysis of blood samples from PD patients, researchers have discovered a significantly higher presence of antimicrobial antibodies [13]. As a result, several infectious agents have been linked to PD [14]. Recent studies have also shown that the intragastric administration of *Listeria monocytogenes* in mice leads to a significant pro-inflammatory response, both locally and systemically, ultimately resulting in neuronal mitochondrial dysfunction. Infected mice showed an increased deposition of aSyn in the gut and elevated levels of p-S129 aSyn levels in the substantia nigra, accompanied by peripheral and neuronal inflammation [15]. 

*Nocardia cyriacigeorgica* is a Gram-positive, rod-shaped, aerobic bacterium belonging to the phylum Actinomycetota (Actinobacteria), and it is commonly found in soil and water. This phylum contains a large number of human pathogens, including mycobacteria, and it is also home to prolific producers of a diverse range of bioactive metabolites, such as antibiotics, signaling molecules, and immunomodulators [16]. Infections by *Nocardia* spp. typically occur through the respiratory tract, although some can also result from the consumption of contaminated food. Previous studies have linked nocardial infections with PD, as an intravenous injection of *Nocardia* spp. in healthy mice resulted in the development of a motor impairment [17]. Furthermore, studies have shown that *N. cyriacigeorgica* accumulates in the brain, particularly in the substantia nigra, which is a crucially affected area in PD, alongside aSyn deposition [18]. As the gastrointestinal system plays a crucial role in PD, we administered *N. cyriacigeorgica* intragastrically to simulate exposure through the consumption of contaminated food or water. 

The role of the gastrointestinal tract in PD has garnered significant interest in recent years. However, the implications of a permeable intestinal barrier in bacterial invasion or infection in PD pathophysiology remain an emerging area of study. A recent study using a chronic low dose of dextran sulfate sodium (DSS) as a colitis model demonstrated several locomotor changes in treated mice [19]. The authors analyzed the mice hippocampus, but no changes were observed. In a study characterizing the DSS-induced colitis model in mice, the authors found that DSS-treated mice had an enlarged spleen [20]. The spleen is responsible for adaptive immunity and the clearing of pathogens [21]. To investigate the impact of colitis on the CNS, previous studies have demonstrated that DSS can have long-term effects on the brain. This is evidenced by an increase in depressive behaviors observed in treated mice [22]. The aim of this study was to further our understanding of PD etiology by subjecting mice to DSS, a well-established colitis inducer in mice, followed by the intragastric inoculation of *N. cyriacigeorgica*. As PD is a disease that develops throughout adult life, we aimed to investigate the impact of time on PD development. To achieve this, we used mice at two different time points: 2 weeks and 12 weeks post-infection. It was observed that DSS, *N. cyriacigeorgica* alone, or the combination of both treatments did not produce a consistent pattern of alterations associated with gut-first disease progression [15,23], namely gut inflammation, aSyn aggregation, mitochondrial fragmentation, intestinal barrier loss, systemic inflammation, and finally, neuroinflammation. These results suggest that gut-first PD pathogenesis requires a cascade of multiple sequential harmful effects to occur in a caudo-rostral direction.

With this work, we aimed to investigate the invasion of opportunistic pathogens into the mammalian body. Also, we sought to understand the mitochondrial dynamics of enteric neurons upon a bacterial infection and the influence of such an event on PD progression.

## 2. Results

### 2.1. DSS Treatment Affects Early-Stage Movement, but Does Not Persist with Age

**Figure 1:**
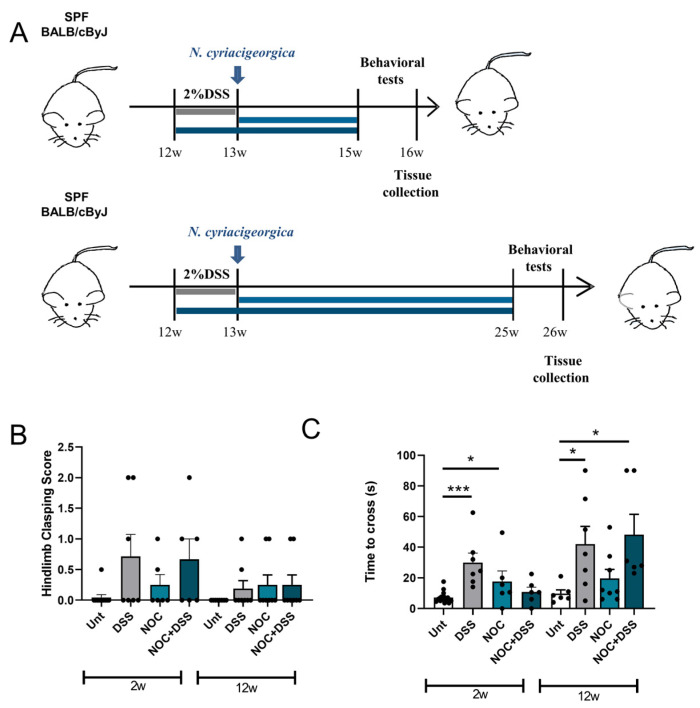
Mice locomotor deficits. (**A**) A schematic representation of the experimental design. The motor behavior was evaluated with (**B**) the hindlimb clasping score and (**C**) the beam-walking test was employed to determine motor coordination. The data represent the mean + SEM. An unpaired Student’s *t*-test was performed in (**B**,**C**). * *p* < 0.05 and *** *p* < 0.001 relative to the corresponding 2 w or 12 w untreated mice.

In mice, intravenous injections of *Nocardia cyriacigeorgica* have been shown to induce locomotion impairments that are responsive to L-DOPA [24]. To investigate the role of the gut in this behavior, we combined a colitis mouse model with an *N. cyriacigeorgica* infection. Twelve-week-old BALB/cByJ male mice were randomly assigned to eight groups, divided into two different time points. For each time point, we had four groups: an untreated group, a group of animals treated with 2% DSS for 7 days, a group treated with *N. cyriacigeorgica* only, and a group treated with 2% DSS for 7 days followed by an oral gavage of bacteria. The initial group of animals was sacrificed two weeks after the intragastric bacterial inoculation, while the second group was sacrificed twelve weeks after the inoculation (Figure 1A). Our data indicate that neither treatment had an effect on motor coordination, as evidenced by the hindlimb clasping test not showing any differences (Figure 1B). However, a tendency was observed for mice treated with DSS or DSS+Nocardia and sacrificed 2 weeks post-infection. In the beam-walking test, mice treated with DSS and those that received the Nocardia treatment 2 weeks post-infection displayed delayed crossing (Figure 1C). After 12 weeks of treatment, DSS and DSS+Nocardia resulted in overt motor impairments (Figure 1C).

### 2.2. Nocardia Administration to a Colitis Model Affects Intestinal Immunity

**Figure 2:**
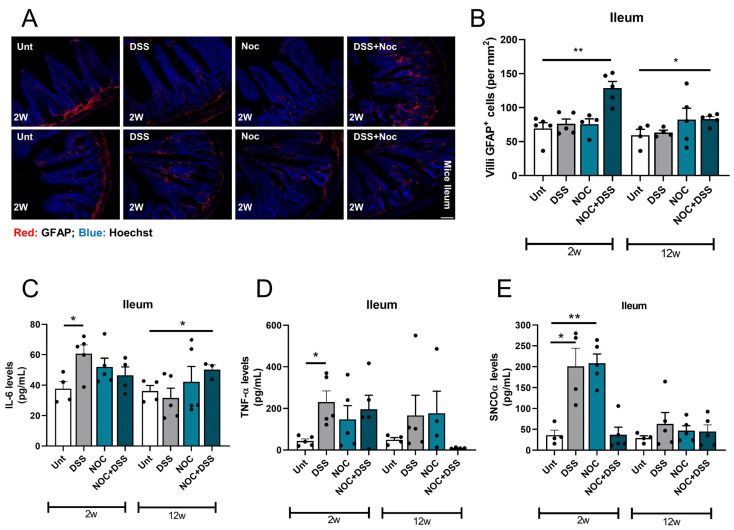
Gut pro-inflammatory environment. (**A**) Representative images of GFAP^+^ cells in ileal transversal slices. (**B**) Quantification of number of GFAP^+^ cells per mm^2^ in villi. IL-6 (**C**) and TNFα (**D**) levels were measured with ELISA kits in ileum lysates to assess gastrointestinal inflammation. (**E**) aSyn levels were measured with ELISA kit in ileal homogenates. Data represent mean + SEM. Scale bar = 50 µm. Unpaired Student’s *t*-test was performed in (**B**–**D**). Ordinary one-way ANOVA was performed in (**E**). * *p* < 0.05 and ** *p* < 0.01 relative to corresponding 2 w or 12 w untreated mice.

Maintaining gut homeostasis requires effective immunity modulation. Therefore, in the context of PD, where immune processes are deeply dysregulated, understanding the impact of compromised immunity on the susceptibility to contracting PD is of enormous interest. To investigate gastrointestinal immunity fitness, we labelled glial fibrillary acidic protein (GFAP)-positive cells in the ileal villi and measured the cytokine levels. It was observed that the mice treated with DSS appeared to have resolved the pro-inflammatory stimulus after two weeks, as the quantity of GFAP^+^ cells was similar to that of the untreated mice. However, the mice that received both treatments displayed increased levels of GFAP^+^ cells (Figure 2A,B). As the mice that received the dual treatment still displayed higher numbers of GFAP^+^ cells 12 weeks post-infection, it can be inferred that this increase is sustained over time. In line with these findings, we noted an increase in the levels of pro-inflammatory cytokine IL-6 in mice treated with DSS for 2 weeks and in those that received the dual treatment and were sacrificed 12 weeks after the infection (Figure 2C). However, the TNFα cytokine levels only increased in the DSS group 2 weeks post-infection. This suggests that, although there was an increased number of GFAP^+^ cells in the intestinal villi, these cells may not have been actively producing TNFα (Figure 2D).

As we had previously observed that a bacterial infection promotes aSyn oligomerization in the gut [15], we sought to investigate whether DSS and nocardial treatments had any effect on the aSyn deposition. Interestingly, we observed an increase in aSyn oligomers in the DSS and Nocardia groups that were sacrificed 2 weeks after treatment (Figure 2E). Recent studies have shown that aSyn is actively involved in inflammatory responses [25], so these results may indicate that aSyn deposition is a short-term response to inflammation.

### 2.3. Fitness of ENS Mitochondria Is Affected by Inflammation

**Figure 3:**
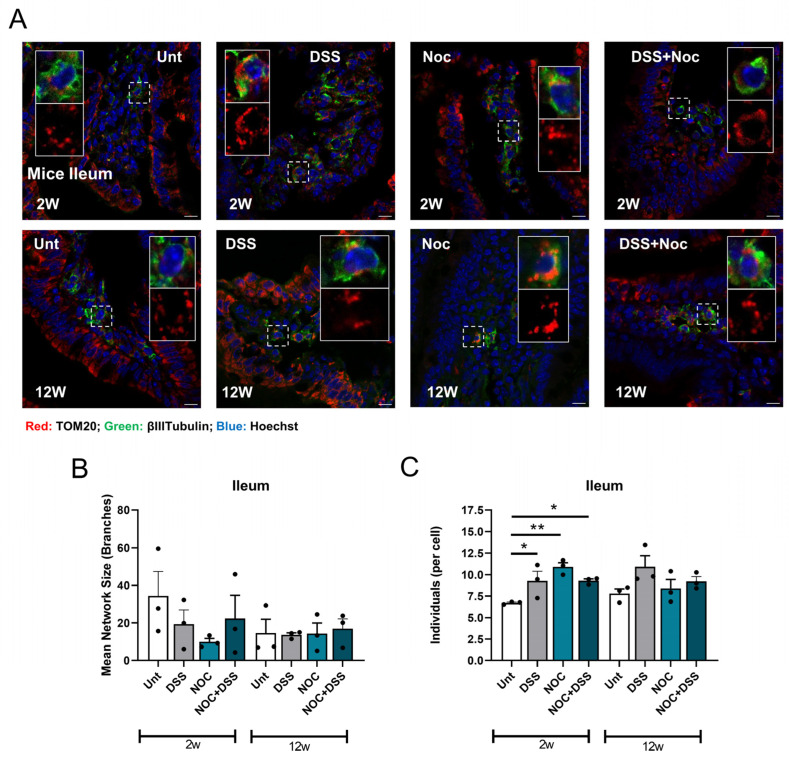
Mitochondrial morphology in enteric neurons. (**A**) Representative images of TOM20/β3-tubulin colocalization in ileum slices. The highlighted square shows a representative enteric neuron stained with β3-tubulin in green and its mitochondrial network in red by staining with TOM20. (**B**) Mitochondrial network and (**C**) number of individual mitochondria were evaluated using the MiNA v100 macro designed for ImageJ (v1.8.0_322). Data represent mean + SEM. Scale bar = 20 µm. Ordinary one-way ANOVA was performed in (**B**,**C**). * *p* < 0.05 and ** *p* < 0.01 relative to corresponding 2 w or 12 w untreated mice.

Mitochondria are essential for the maintenance of viable enteric nervous system (ENS) neurons, and thus, they influence gut homeostasis. It has been observed that PD patients have aberrant mitochondria in the ENS [26]. In addition, a study in which rotenone—a complex I inhibitor of the electron transport chain—was administered intragastrically to mice found that it induced an increase in the levels of aSyn aggregates [27]. We therefore investigated how the mitochondrial function was affected by the above treatments. Our results showed that, although the mean network size of the mitochondrial network of enteric neurons remained unaltered (Figure 3A,B), the DSS-, Nocardia-, and DSS+Nocardia-treated mice sacrificed 2 weeks after the infection had a reticulated mitochondrial network (Figure 3C), suggesting that intestinal inflammation and bacteria are able to modulate mitochondrial function.

### 2.4. Gut Permeability and Systemic Inflammation

**Figure 4:**
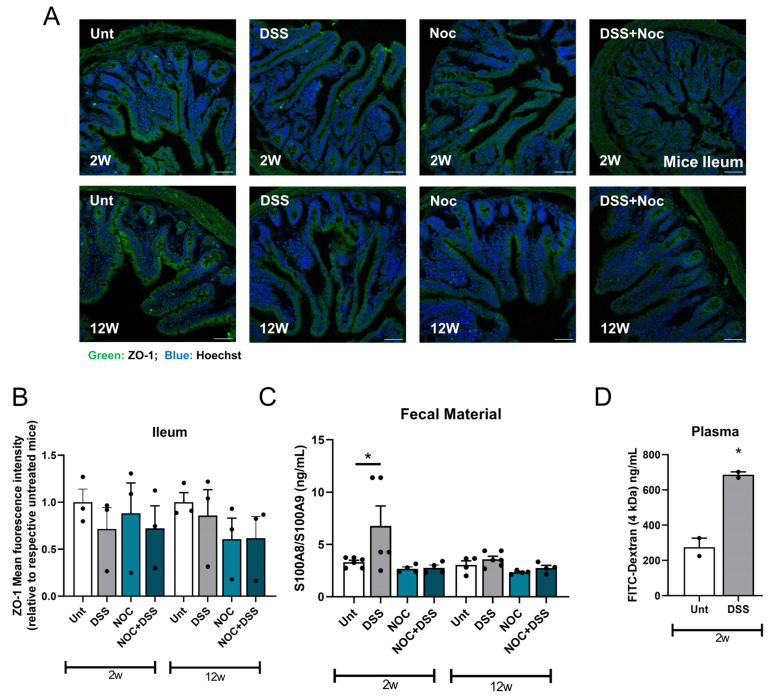

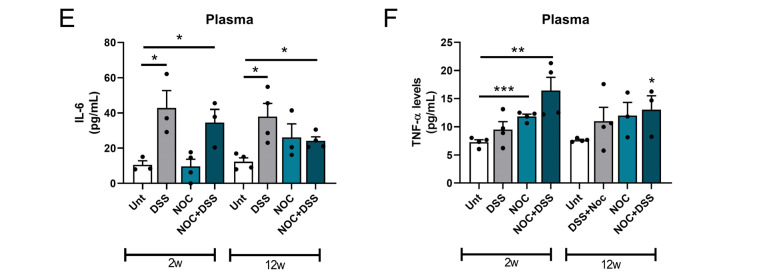
Gut permeability and systemic inflammation. (**A**) Representative images of ZO-1 expression in ileal transversal slices. (**B**) Mucosal cohesion was evaluated by determining mean gray values of ZO-1 signal. (**C**) Calprotectin was measured with an ELISA kit in fecal material obtained from mice. (**D**) FITC-Dextran fluorescence was measured in harvested blood against a concentration curve. (**E**) IL-6 and (**F**) TNFα levels were measured with ELISA kits in mice plasma. Data represent mean + SEM. Scale bar = 50 µm. Ordinary one-way ANOVA was performed in (**B**,**C**,**E**,**F**). Unpaired Student’s *t*-test was performed in (**D**). * *p* < 0.05, ** *p* < 0.01 and *** *p* < 0.001 relative to the corresponding 2 w or 12 w untreated mice.

The gastrointestinal tract is the first line of defense against pathogens and foreign substances [28]. Therefore, the cohesion of the gastrointestinal mucosa is essential to keep the organism free from such harmful agents. As the gastrointestinal mucosa is constantly being renewed, intestinal epithelial cells easily adhere to each other with the help of tight junctions. ZO-1 is a key tight junction protein that helps maintain the intestinal permeability at a healthy level [29]. We therefore analyzed the robustness of this tight junction protein by immunofluorescence. Our data show that intestinal inflammation did not alter the localization or levels of this protein (Figure 4A,B). Nevertheless, we observed that DSS induced a leaky gut after 2 weeks of treatment, as calprotectin, a protein secreted by neutrophils, was present in the fecal material. This protein is a biomarker of intestinal barrier permeability and inflammation and it has also been found to be elevated in PD patients [30]. The results of our experiments show that no differences were observed in the fecal material of the mice in any of the other groups (Figure 4C), which is consistent with the previous observations on TNFα. To validate our previous data and to check that the DSS treatment was carried out correctly, we administered FITC-dextran by gavage to DSS-treated mice to assess their intestinal permeability. In the case of intestinal permeability, this compound enters the blood, where it is detected. The 2-week DSS group showed a clear increase in gut permeability (Figure 4D), confirming the success of the DSS treatment.

We further investigated the systemic inflammatory status of the mice by determining the levels of pro-inflammatory cytokines in the blood. The plasma levels of IL-6 increased after the DSS and DSS+Nocardia treatments at 2 and 12 weeks (Figure 4E). TNFα increased in the 2-week post-infection groups, in the mice treated with Nocardia, and also in the mice receiving the double treatment (Figure 4F). Mice sacrificed at 12 weeks post-infection showed an increase in their blood TNFα levels after the DSS+Nocardia treatment (Figure 4F). These results suggest that DSS-induced intestinal barrier leakage may be transient, since it seems to require other triggers, such as a Nocardia infection, to continue.

### 2.5. Midbrain Immunity Activation after Gut Inflammation

**Figure 5:**
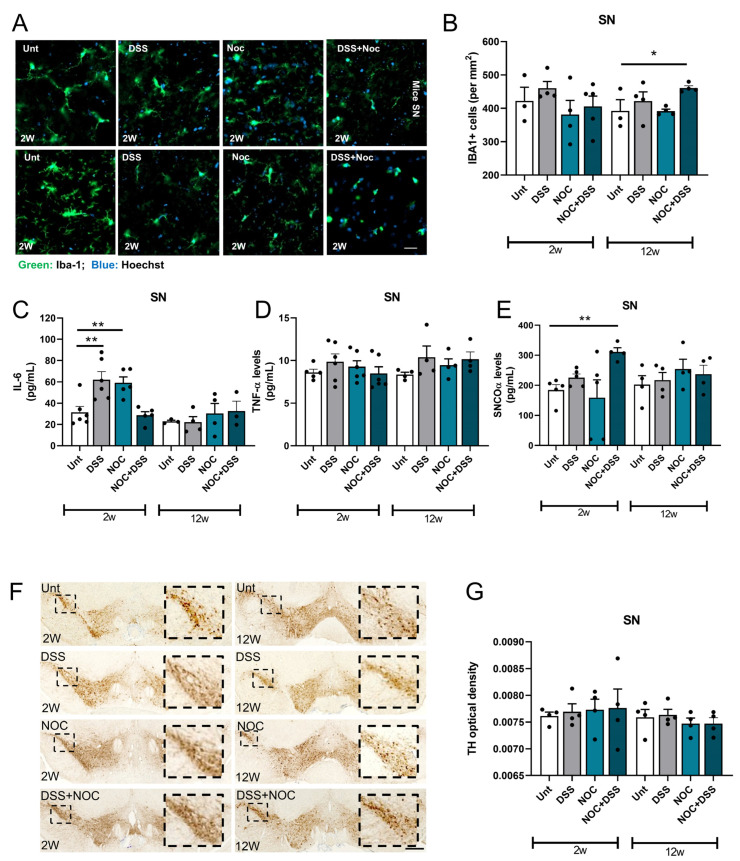
Midbrain PD features. (**A**) Representative images of IBA-1 staining in substantia nigra region in brain coronal sections. (**B**) Number of IBA-1^+^ cells per mm^2^ in substantia nigra. (**C**) IL-6 and (**D**) TNFα cytokine levels were measured with an ELISA kit. (**E**) aSyn oligomers were measured using an ELISA kit. (**F**) Representative images of TH staining in substantia nigra. The highlighted square shows the area selected in the small square with increased magnification. (**G**) Optical density of TH staining. Data represent mean + SEM. Scale bars = (**A**) 20 µm and (**F**) 300 µm. Ordinary one-way ANOVA was performed in (**B**–**E**,**G**). * *p* < 0.05 and ** *p* < 0.01 relative to corresponding 2 w or 12 w untreated mice.

We have previously shown that gut dysbiosis is sufficient to induce a pro-inflammatory environment in the gastrointestinal system, triggering systemic and brain inflammation [23]. We therefore investigated how gut inflammation induced by the DSS+Nocardia treatment triggered neuroinflammation. By examining brain slices for the presence and activation of microglial cells, we showed that mice at 12 weeks post-infection that received both the DSS and Nocardia treatments had an increased number of IBA-1^+^ cells in the substantia nigra, which may indicate the beginning of a neuroinflammatory process. Since mitochondria are involved in the activation of innate immunity [31] and can induce a pro-inflammatory response, we sought to observe how mitochondria-dependent neuroinflammation was modulated and whether there was any involvement in aSyn deposition. We did not observe changes to the mitochondrial network fragmentation in midbrain dopaminergic neurons in any of the experimental groups (Figure S1). Nevertheless, the DSS- and Nocardia-treated mice sacrificed 2 weeks after treatment showed higher levels of midbrain IL-6 (Figure 5C), although no changes were observed in the mice who received the dual treatment. Our data also show that no differences in TNFα levels were observed in any of the experimental groups (Figure 5D). In contrast, it appears that, although mitochondria-dependent inflammation did not occur in mice 2 weeks after receiving the double treatment, an increase in aSyn deposits occurred in the midbrain (Figure 5E).

SNpc dopaminergic neurons are the first to deteriorate in PD patients. These cells are a distinct group of cells that have high levels of tyrosine hydroxylase (TH), an enzyme that is crucial for dopamine production. We therefore tested for neurodegeneration by labelling midbrain slices with anti-TH antibodies, but no differences were observed among the experimental groups (Figure 5F,G). The combination of these results allowed us to conclude that, although no obvious changes in midbrain dopaminergic neurons were observed, the mice treated with both DSS and Nocardia may have already been in the prodromal phase of PD.

## 3. Discussion

Over the last decade, the gut–brain axis has become increasingly important in the pathophysiology of PD. The GI tract is essential for immune modulation and maturation because it is an organ that comes into contact with many substances that are exogenous and unfamiliar to the mammalian organism. Recent studies from our laboratory have shown that the targeting of mitochondria with a microbial toxin or with bacteria disrupts mitochondrial homeostasis in the gut, triggers an inflammatory cascade, and favors aSyn aggregation. In addition, in both cases, the mice developed significant motor impairments [15,23]. In the present study, we sought to further investigate the effect of a pro-inflammatory challenge in the pathophysiology of the disease using a colitis model prior to an intragastric inoculation with *Nocardia cyriacigeorgica*, a bacterium known to induce parkinsonian features in injected mice. Although intravenous injections have provided insight into the nocardial tropism for the substantia nigra and correlated it with PD through motor defects, this procedure clearly bypasses the main barrier to pathogens in the mammalian system and does not mimic a natural infection process, nor is it useful for determining the fundamental role of the gut in the etiology of gut-first PD. Our results show that animals sacrificed 2 weeks after the cessation of the DSS treatment and mice infected with Nocardia had a deficiency in crossing the beam, which may have been due to some sickness behavior that may have still been occurring in the colitis and bacteria group. After 12 weeks of treatment, the combined effect of both DSS and bacteria produced a further effect on the motor function of mice. Previous studies with Nocardia have elegantly demonstrated a variety of physical impairments [17,24]. However, an intravenous injection does not recapitulate a natural process and facilitates undesirable bacterial dissemination throughout the body. In this regard, we also observed that the intragastric administration of bacteria does not induce a consistent motor defect at either time point, suggesting a more difficult pathway to neurodegeneration.

Enteric glia are specialized cells that support the immune system of the gastrointestinal tract and are the major producers of TNFα upon infection [32,33,34]. Although we observed an increase in the number of GFAP-positive cells in the group that was treated with both DSS and Nocardia, we only observed an increase in the IL-6 levels after 12 weeks. The TNFα levels only increased after 2 weeks of the DSS treatment. The intestinal barrier permeability plus the Nocardia infection with higher levels of GFAP-positive cells may indicate that the infection delayed ileal healing, i.e., prolonged the period of inflammation.

aSyn deposition occurs in response to inflammation in the gut [35]. While there was a peak in aSyn deposition immediately after the DSS and Nocardia treatments, the combination of these treatments did not result in an increase in aSyn oligomers in the gut. This appears to be a contrasting result that requires further investigation. At 12 weeks post-treatment, no difference in aSyn deposition was observed in any of the experimental groups, suggesting that the mice were able to clear aSyn oligomers after an acute pro-inflammatory stimulus. This suggests that aSyn oligomerization can occur due to pro-inflammatory challenges in the gut and that aSyn oligomers can be effectively cleared [35].

Mitochondria are essential players in innate immunity, particularly through the NLRP3 inflammasome cascade, which promotes the production and release of pro-inflammatory cytokines [36]. In the gut-first PD hypothesis, enteric neurons also gain importance, mainly for the putative retrograde transport of aSyn to the CNS [23,37]. Therefore, we observed the mitochondrial fitness of enteric neurons by immunofluorescence. We detected a higher number of mitochondrial individuals in each treatment, but only after 2 weeks. As there were no differences in the mice 12 weeks after treatment, it seems reasonable to conclude that the mitochondrial turnover can be replenished, which seems to be related to the clearance of aSyn oligomers. Indeed, we already showed that, after an infection, aSyn accumulates inside the mitochondria, creating a positive feedback loop that potentiates neuronal innate immune activation and, thus, inflammation [15,38].

In mammals, mucosal cohesion is conferred by the expression of tight junctions in the gastrointestinal tract [39]. It has also been observed that intestinal permeability allows for the invasion of bacteria or metabolites into the intestinal epithelial environment, including the enteric nervous system [40,41]. Thus, a local pro-inflammatory agent could influence systemic inflammation. Although we did not detect differences in the intestinal ZO-1 levels or localization, DSS was able to induce intestinal barrier permeability, which resolved after 12 weeks of treatment. Nevertheless, our results suggest that the DSS plus Nocardia initial treatments produced a pro-inflammatory outburst capable of inducing a long-lasting increase in the systemic cytokine levels.

Neuroinflammation is at the core of PD pathophysiology, and has a detrimental effect on disease progression [42]. Interestingly, we found that the only group that showed microglial activation was the group that received both DSS and Nocardia treatments and was sacrificed 12 weeks after treatment. This group showed intestinal and systemic inflammation. This result supports the observation that systemic inflammation occurs in the early stages of the treatments, with implications for later stages. Interestingly, the group of mice that received DSS plus Nocardia and were sacrificed 2 weeks after the treatment showed higher levels of aSyn oligomers in the midbrain, despite no increase in pro-inflammatory cytokines or microglial activation. In addition, no changes in TH density were observed in any group of the experiment.

## 4. Materials and Methods

### 4.1. Key Resources Table

The sources of reagent or resources used in this study are listed below (Table 1).

### 4.2. Animal Model and Experimental Design

Male 12-week-old BALB/cByJ mice were maintained in a tightly controlled environment with a 12 h–12 h light/dark cycle under ABSL2 conditions, with a regulated temperature (20–21 °C) and humidity (45–55%). Water and standard chow were provided ad libitum throughout the experiments. The mice were randomly assigned to the control (untreated) and treatment groups. The animals were continuously monitored for signs of distress throughout the treatments. A loss of >20% body weight was defined as the humane endpoint for the study, in accordance with EU and Portuguese legislation (Directive 2010/63/EU; DL113/2013, August 7th) for the care and use of animals. All treatments and procedures were performed in accordance with the ethical guidelines of the Animal Welfare Committee of the Center for Neuroscience and Cell Biology and the Faculty of Medicine, University of Coimbra (ORBEA and DGAV, 30 June 2017). The researchers were appropriately trained in the procedures involving animals (FELASA certified course) and certified by the Portuguese authorities (Direção Geral de Veterinária).

### 4.3. Bacteria and Infection

*Nocardia cyriacigeorgica* strain IMMIB D-1627 was obtained from the German Collection of Microorganisms and Cell Cultures GmbH and cultured overnight in brain heart infusion (BHI) broth at 35 °C with shaking (120 rpm). To remove clumps of bacteria, the inoculum was centrifuged for 10 min at 100× *g*. The bacteria were then washed twice with sterile 1 × PBS and suspended in PBS. The mice were inoculated with 10^7^ CFU/mL of the bacterium by oral gavage and untreated animals were gavaged with sterile 1 × PBS. The mice were monitored to ensure that no harm was caused by the oral gavage and were left undisturbed until sacrificed.

### 4.4. Behavior

For motor coordination and balance, we performed the beam-walking and hindlimb-clasping tests. For beam walking, we tested the ability of mice to cross a narrow 1 m beam and reach a platform on the opposite side of the beam. The mice were allowed to explore the platform for 30 s to familiarize themselves with the test in a squared beam. The mice then performed 2 trials on an 8 mm round beam, with a maximum of 90 s to complete each trial. The mice that did not fully cross the beam were given a 90 s value for the trial. At least 1 h was provided between trials to allow the mice to rest.

The hindlimb-clasping score was used to assess neurodegeneration in mice. The mice were suspended by their mid-tail for 10 s and each test was scored from 0 to 3 based on hindlimb movement as described elsewhere [43]. To ensure more accurate results, the animals were randomly assigned and at least 2 researchers were blinded to the mouse conditions.

### 4.5. Immunohistochemistry

For immunohistochemistry, the mice were anesthetized and transcardially perfused with PBS, followed by a 4% PFA perfusion for tissue fixation. The harvested organs were maintained in 4% PFA overnight, switched to serial solutions of 15% and 30% sucrose, and then embedded in Tissue-Tek^®^ O.C.T. (Sakura Finetek, Torrance, CA, USA). The organs were stored at −80 °C until further use. Brain and ileum samples were sliced with a Epredia™ CryoStar™ NX50 Cryostat (Fisher Scientific, Hampton, NH, USA) in 20 µm slices and stored. At the time of use, the slices were thawed and hydrated with 1 × PBS for 30 min, washed twice in PBS for 5 min, and blocked with a 10% goat serum/0.25% Triton X-100 solution for 1 h. The slices were then washed three times with PBS for 5 min each and primary antibodies (GFAP, Santa Cruz (cat. No. sc-71143); TOM20, Santa Cruz (cat. No. sc-11415); β3-tubulin, Cell Signaling (cat. No. #4466); ZO-1, Abcam (cat. No. ab96587); IBA-1, FUJIFILM Wako Chemicals (cat. No. 019-19741); and TH, Millipore (cat. No. AB152)) were incubated overnight at 4 °C in 1% goat serum. The slices were then brought to RT, washed three times with PBS, and incubated with secondary antibodies for 2 h at RT. After two washes with PBS, the slices were incubated with Hoechst 33342 for 15 min to label the nuclei, mounted with Mowiol 4-88 (Sigma, St. Louis, MO, USA), and sealed with nail polish.

### 4.6. ELISA Determination of Pro-Inflammatory Cytokines and aSyn and Calprotectin Fecal Levels

ELISA was performed to assess the pro-inflammatory cytokine levels and aSyn oligomerization. For this purpose, mouse blood was collected by a cardiac puncture just before perfusion, both the ileum and the brain were harvested, and the protein content was extracted using a lysis buffer containing protease and phosphatase inhibitors. ELISA kits for IL-6, TNFα, and aSyn were used according to the manufacturers’ instructions. ELISA was performed to detect the calprotectin levels in mouse fecal material. The fecal material was collected at the end of the experiment and the calprotectin levels were determined with an ELISA kit according to the manufacturer’s instructions.

### 4.7. Microscopy

Images for inflammatory markers in ileal samples and for microglia visualization in the brain were obtained using a LSM 710 confocal microscope (Zeiss; Oberkochen, Baden-Württemberg, Germany) with a Plan-Apochromat 40×/1.4 Oil DIC M27 objective at 512 × 512 resolution. For GFAP and ZO-1 in the intestinal villi, at least 15 villi per animal were analyzed in at least three different sections. For GFAP, z-stack images were flattened and the number of cells was counted and divided by the villus area, while for ZO-1 integrity, the mean gray values were evaluated. To evaluate enteric neuron mitochondria, we performed a colocalization between TOM20 and β3-tubulin in the ileal samples. The β3-tubulin signal was masked and multiplied by TOM20 flattened images to ensure that only the enteric neuron mitochondria were evaluated. The resulting images were analyzed using the MiNA V100 macro developed for ImageJ (v1.8.0_322). For the visualization of Iba-1 in the substantia nigra, six different stereological areas per animal were analyzed in three different sections. Z-stacks were flattened to average the projection images, the number of Iba-1+ cells was counted, and the values were divided by the field of view area. To determine the TH density in the mouse substantia nigra, images were acquired using a Carl Zeiss Axio Scan Z1 (Zeiss) slide scanner with a Plan Apochromat 20×/0.8 Air. A minimum of six different stereological areas were analyzed per animal in at least three different sections. The stereological area corresponding to the substantia nigra was outlined and the optical density of the area was determined.

### 4.8. Intestinal Permeability Assay

To evaluate intestinal permeability, we performed the fluorescein isothiocyanate-dextran (FITC-dextran) assay. To this end, mice were fasted for 6 h and received 150 µL of FITC-dextran (4 kDa) at a concentration of 80 mg/mL in sterile PBS by oral gavage. The mice were left undisturbed for 4 h and then euthanized for blood collection. The blood was then centrifuged at 400 rpm for 20 min at RT and the plasma collected was probed for FITC-dextran fluorescence. The fluorescence was measured at 530 nm with excitation at 485 nm.

## 5. Conclusions

In conclusion, this study shows that, like what occurs in the prodromal phase of PD, an inflammatory process was underway in these mouse guts, but the pathology only progressed to the CNS with the loss of dopaminergic neurons in the midbrain if several other processes occurred sequentially. We hypothesize that, after a trigger, in our case, DSS and a Nocardia infection, facilitators such as the loss of the intestinal barrier, the fragmentation of the mitochondrial network, and the aggregation of aSyn are required to allow aggravators, such as neuroinflammation, to induce the degeneration of dopaminergic neurons [44].

## Figures and Tables

**Table 1:** Key resources.

Reagent or Resource		Source
Antibodies—IHC/IF		
Mouse anti-GFAP		Santa Cruz Biotechnology (Dallas, TX, USA; cat. No. SC-71143)
Rabbit anti-ZO-1		Abcam (Cambridge, UK; cat. No. ab96587)
Rabbit anti-tyrosine hydroxylase (TH)		Millipore (Burlington, MA, USA; cat. No. AB152)
Rabbit anti-Iba1		FUJIFILM Wako Chemicals (Neuss, Germany; cat. No. 019-19741)
Mouse anti-β3Tubulin		Cell Signaling (Danvers, MA, USA; cat. No. 4466)
Rabbit anti-Tom20		Santa Cruz Biotechnology (Dallas, TX, USA; cat. No. SC-11415)
Biotinylated anti-rabbit IgG		Vector Labs (Newark, CA, USA; cat. No. BA-1000)
Goat anti-mouse Alexa Fluor 488		Molecular Probes, Life Technologies (Waltham, MA, USA; cat. No. A11001)
Goat anti-mouse Alexa Fluor 594		Molecular Probes, Life Technologies (Waltham, MA, USA; cat. No. A11005)
Goat anti-rabbit Alexa Fluor 594		Molecular Probes, Life Technologies (Waltham, MA, USA; cat. No. A-11012)
Goat anti-rabbit Alexa Fluor 488		Molecular Probes, Life Technologies (Waltham, MA, USA; cat. No. A11008)
**Chemicals**		
Hoechst		Invitrogen (Waltham, MA, USA; cat. No. H1399)
FITC-dextran sulfate sodium salt		Sigma-Aldrich (St. Louis, MO, USA; cat. No. 78331)
DPX mountant		Sigma (St. Louis, MO, USA; cat. No. 06522-100 mL)
Vectastain Elite ABC Perox standard kit		Vector Labs (Newark, CA, USA; cat. No. VCPK-6100)
Normal goat serum		Abbkine (Georgia, USA; cat. No. BMS0050)
MOM blocking reagent		Vector Labs (Newark, CA, USA; cat. No. MKB-2213-1)
OCT mounting medium		Carl Roth (Karlsruhe, Germany; cat. No. KMA-0100-51A)
**Kits**		Sensitivity
Mouse IL-6 ELISA kit	R&D Systems (Minneapolis, MN, USA; cat. No. M6000B)	1.3–1.8 pg/mL
α-Synuclein oligomer (SNCO α) ELISA kit	MyBioSource (San Diego, CA, USA; cat. No. MBS724099)	1.0 pg/mL
Calprotectin kit	Invitrogen (Waltham, MA, USA; cat. EM67RB)	0.65 ng/mL
Mouse TNF-α Quantikine ELISA	R&D Systems (Minneapolis, MN, USA; cat. No. MTA00B)	0.36–7.21 pg/mL

## Data Availability

Data is contained within the article and Supplementary Materials.

## Supplementary Materials

The following supporting information can be downloaded at: https://www.mdpi.com/article/10.3390/ijms25063423/s1.
