# Supplementary material for: Nocardia cyriacigeorgica Elicits Gut Disturbances in a Leaky Gut Model of Colitis, but Not the Harmful Cascade Leading to Gut-First Parkinson’s Disease
Source: Int J Mol Sci. 2024 Mar 18;25(6):3423. doi: 10.3390/ijms25063423 (PMC10970553; doi:10.3390/ijms25063423)
Supplement: Supplementary file 1 [file ijms-25-03423-s001.zip › ijms-2864966-supplementary.pdf]

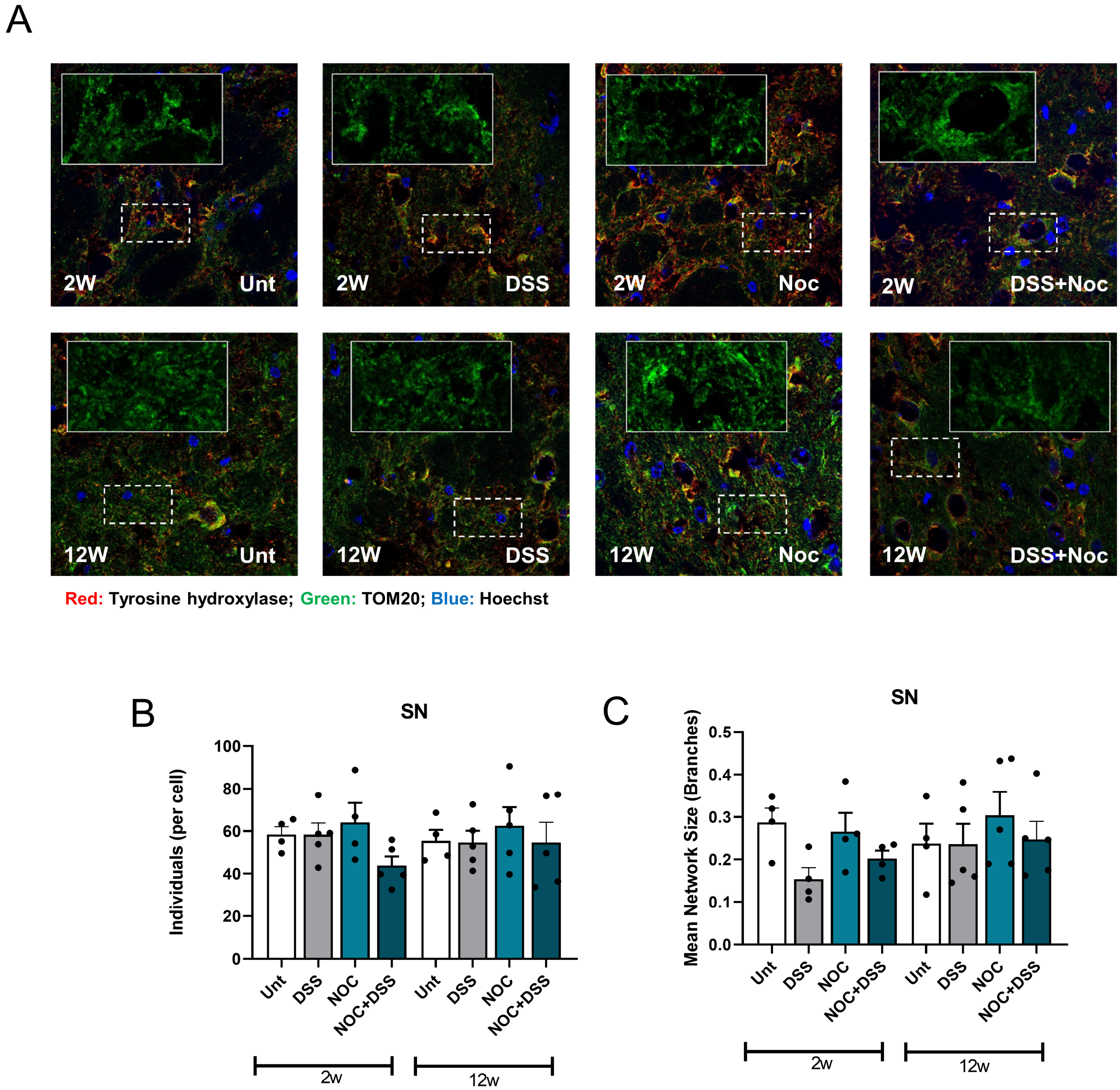

Supplemental Figure S1. Mitochondrial morphology in midbrain neurons. (**A**) Representative images of TOM20/β3-tubulin colocalization in ileum slices. (**B**) Mitochondria network and (**C**) number of individual mitochondria were evaluated using the MiNA v100 macro designed for ImageJ. Data represents mean + SEM. Scale bar = 20 μm. Ordinary one-way ANOVA was performed in (**B**,**C**), relative to the corresponding 2w or 12w untreated mice.
